# High Resolution Synchrotron X-Radiation Diffraction Imaging of Crystals Grown in Microgravity and Closely Related Terrestrial Crystals

**DOI:** 10.6028/jres.096.017

**Published:** 1991

**Authors:** Bruce Steiner, Ronald C. Dobbyn, David Black, Harold Burdette, Masao Kuriyama, Richard Spal, Lodewijk van den Berg, Archibald Fripp, Richard Simchick, Ravindra B. Lal, Ashok Batra, David Matthiesen, Brian Ditchek

**Affiliations:** National Institute of Standards and Technology, Gaithersburg, MD 20899; EG&G Energy Measurements, Goleta, CA 93116; NASA Langley Research Center Hampton, VA 23665; Alabama A&M University, Huntsville, AL 35762; GTE Laboratories, Waltham, MA 02254

**Keywords:** crystal growth, diffraction, gallium arsenide, high resolution, lead tin telluride, mercuric iodide, microgravity, synchrotron, topography, triglycine sulfate

## Abstract

Irregularities in three crystals grown in space and in four terrestrial crystals grown under otherwise comparable conditions have been observed in high resolution diffraction imaging. The images provide important new clues to the nature and origins of irregularities in each crystal. For two of the materials, mercuric iodide and lead tin telluride, more than one phase (an array of non diffracting inclusions) was observed in terrestrial samples; but the formation of these multiple phases appears to have been suppressed in directly comparable crystals grown in microgravity. The terrestrial seed crystal of triglycine sulfate displayed an unexpected layered structure, which propagated during directly comparable space growth. Terrestrial Bridgman regrowth of gallium arsenide revealed a mesoscopic structure substantially different from that of the original Czochralski material. A directly comparable crystal is to be grown shortly in space.

## 1. Introduction

The performance of electro-optic devices varies according to the crystal growth and fabrication procedures used; and increases in the ability to control these procedures now promise substantial improvement in such devices. In particular, reduction of convection in the microgravity found in space now offers control of one very important parameter in crystal growth. Nevertheless, the absence of comprehensive knowledge of the principal structural defects engendered during the various stages of crystal growth and device fabrication and of the roles played in device performance by the various defects has severely restricted device improvement to date.

In order to begin to shed light on the principal irregularities found in various electro-optic detector materials and their influence on device performance, we have observed and compared irregularities found in three crystals grown in space and in four *directly comparable* crystals grown on the ground. These irregularities, which were observed in mercuric iodide, lead tin telluride, triglycine sulfate, and gallium arsenide by high resolution synchrotron x-radiation diffraction imaging provide new clues to the nature and origins of the principal irregularities in these important materials and on their respective influence on detector performance.

Detector materials are of special interest because the performance of detectors made from space-grown mercuric iodide has been reported to be far superior to similar devices made from similar ground-grown material. The charge carrier mobility of x-and gamma-ray detectors made from space-grown crystals was at least six times higher than for similar detectors made from ground-grown crystals [1] [2]. This is expected to lead to increased energy resolution in the radiation detectors made from this material. Determination of the principal irregularities in these materials is of interest: 1) for the scientific insight to which it can lead, 2) for the optimization of expensive space growth, and ultimately 3) for the establishment of desirable growth conditions in far less extreme and expensive environments on the basis of the insight achieved.

Establishment of the specific *nature* of the mesoscopic irregularities in these materials, determination of their *level of incidence*, and observation of their *distribution* in space-grown and comparable terrestrial materials are all important to these goals. Fortunately, recent technical advances in diffraction imaging with highly parallel monochromatic synchrotron x-radiation present the first opportunity for crystal growers to obtain all of these parameters simultaneously [3, 4, 5].

## 2. Imaging Goals

### 2.1. General Considerations

X-ray topography alone has long promised to provide this information simultaneously on the *nature, prevalence*, and *distribution* of structural irregularities over the macroscopic areas important to integration with crystal growth parameters. However, this information clearly has not been available. A principal impediment to the fulfillment of this promise has been x-ray beam divergence. Individual irregularities and the immediately surrounding matrix in a typical crystal are illuminated by laboratory x-ray sources over an angular range measured in arc minutes. The divergence of this incoming beam (at a given point on the sample) unfortunately supports diffraction simultaneously from many irregularities along with that from the surrounding regular regions. The spread in wavelength in white beam synchrotron radiation also permits diffraction simultaneously from irregular and regular regions. In both instances, contrast that would otherwise be present is severely reduced or eliminated entirely. Even where some contrast remains, the spatial information contained is convoluted by the differing Bragg angles in a way that will not permit unfolding and subsequent detailed analysis.

Lattice deviations influencing diffraction by seconds of arc, which are frequently critical to satisfactory interpretation and understanding of mesoscopic irregularity, are rendered visible in diffraction only by a source of monochromatic radiation parallel within an arc second. In such a beam, spatial fidelity also is preserved at the micrometer level. The recent availability of such a source of x-radiation thus now permits the realization of the long term promise of x-ray topography. Among the irregularities that can now be observed *over areas large enough to interpret in terms of crystal growth* are the following.

### 2.2 Lattice Orientation and Strain

Of the various types of irregularity in high-quality crystals, perhaps the most pervasive is gradual change in the lattice. The orientation of the lattice or the magnitude of the lattice parameter, or both, may vary. For any one orientation of the crystal with respect to the incident beam, such variation results in diffraction only from a portion of a single grain.

Diffraction from grains whose *lattice orientation or parameter varies monotonically or aperiodically* yields images of restricted regions of a single grain. The part of such a grain that is in diffraction shifts gradually as the crystal is rotated. The moving edge of this image is characteristically soft and relatively indistinct in high resolution diffraction images.

In other systems, such as the Czochralski growth of doped material, *lattice variation* may be *oscillatory*, leading to *striations* in diffraction images of crystals cut obliquely to the local growth direction and oriented slightly off of the Bragg condition. Contrast is inverted on opposite sides of the Bragg diffraction peak. These striations record, like tree rings, not only variation in chemical composition but, taken together, also the *shape of the crystal at various stages in its growth*; and they can be deciphered in a somewhat similar if more complex and sophisticated manner [5, 6].

### 2.3 Grain and Subgrain Boundaries

Sharp contrast in the image of a crystal can delineate homogeneous grains or subgrains. In contrast to the preceding case, the boundaries of such an image do not move as the crystal is rotated in the Bragg direction. Where the lattice orientation of a pair of such homogeneous grains differs by rotation in the diffraction plane by more than the acceptance angle, only one of these grains (or subgrains) will diffract at a time; and, if it is not strained, it does so in its entirety. Variations in real-time images of such a crystal permit rapid and detailed assessment of the *relative misorientation* (in the Bragg direction) *of the various grains and subgrains* with respect to one another.

Where the lattice orientation of a pair of such contiguous grains differs in a direction orthogonal to the plane of diffraction, both grains may appear in diffraction simultaneously, but the resulting images are displaced with respect to one another in this direction. The pair of these images is either separated or overlapped, depending on the relative inclination of the two lattices.

### 2.4 Dislocations

Dislocations typically appear in diffraction images taken in Laue geometry (transmission) as linear features that are broader at one end than the other. The broadening of one end of such a feature arises from scattering deep within the crystal, while the sharp end locates the intersection of the dislocation with the x-ray exit surface of the crystal. The *orientation of a dislocation* can be determined with high precision for those cases in which the intersection of the dislocation with both entrance and exit faces is distinct in the diffraction image.

Variation in the visibility of such a line feature in successive diffraction images indicates the *direction of atomic displacement associated with a dislocation*, which is parallel to its Burgers vector. However, since the visibility of such a dislocation varies relatively slowly, that is, as the cosine of the angle between the Burgers vector and the diffraction vector, the determination of this direction is most precise when contrast can be observed to disappear at one unique angle. When the direction of diffraction is oriented normal to the atomic displacement, such a feature vanishes from the diffraction image.

### 2.5 Phase Domain Boundaries

*Twins* are distinguished normally by absence of diffraction from regions between sharp parallel boundaries visible in some diffraction directions but not in others. The contrast in the latter when observed by high-resolution beams may be affected by very slight differences in lattice alignment.

With angular collimation of the order of an arc second, the images of other boundaries recorded in Laue geometry may also become visible when the diffraction vector falls along the boundary. Such boundaries are visible even under these restrictive conditions only when they separate atomically coherent regions differing by an atomic phase shift [7,8]. Those boundaries that have been observed to date to fulfill these conditions appear to separate *antiphase domains.* Radiation with a divergence of the order of a second of arc or less is necessary to image such boundaries.

### 2.6 Additional Phases

The absence of diffraction from particular regions of a crystal under all diffraction conditions supporting diffraction from the rest of the crystal strongly suggests the presence of a *second phase*, although in principle the non diffracting regions may simply be misoriented with respect to the rest of the crystal. In stoichiometric materials, the boundaries of two phases are sharply delineated. In alloys, this sharpness is vitiated by the gradual changes in composition that may be permitted.

### 2.7 Surface Scratches

The strain associated with surface scratches is linear, but sometimes gently curved, and typically non crystallographic in orientation. They are typically distinguished also by three other characteristics: 1) uniform width, 2) sharp edges, and 3) contrast reversal either laterally, longitudinally, or both, particularly as the Bragg peak is scanned. The latter two characteristics are evident both in observation in Bragg geometry and when scratches are present on the exit surface in Laue geometry.

## 3. Current Imaging Capability

Suitable synchrotron radiation sources now offer opportunities to fulfill the long awaited promise of x-ray topography; but the degree of success in their realization depends upon the particular parameters of the storage ring and its beam lines. Since the precise orientation of the x radiation at individual points on the sample is crucial to the analysis, the vertical source size, together with the distance of the sample from the tangent point on the ring, may limit the utility of the images produced.

The x-ray storage ring of the National Synchrotron Light Source at Brookhaven National Laboratory offers the most suitable combination of characteristics of any existing storage ring, providing an unusually bright beam whose degree of vertical divergence at a point on a sample mounted on beam line X-23A3 is 1.5 arc seconds.

Although this 1.5 arc second beam provides a considerable improvement over other sources, it is not yet sufficient for diffraction imaging with optimum sensitivity to defects. For useful sensitivity to irregularities, which requires further improvement in beam divergence by another order of magnitude, i.e., 0.1 arc second, the optics of the monochromator are crucial. Such a beam is necessary for rendering critical features visible, for preservation of the spatial information in the image within the plane of diffraction, and for displaying essential clues to the strains upon which the success or failure of detailed analysis can depend [6].

With such a dedicated 0.1 arc second monochromatic capability, however, which is available on a routine basis only on Beam Line X-23A3 at the National Synchrotron Light Source, the detection and interpretation of irregularities are limited principally by their density. Irregularities can be recorded photographically with a spatial resolution of 1 μm. Observation with an x-ray vidicon and charge coupled device (ccd) cameras readily provides complementary information with a spatial resolution of 35 μm with intermediate sensitivity in real-time, and 20 μm with shot noise limited sensitivity in quasi real-time.

## 4. The Crystals

Three crystals of mercuric iodide, two of lead tin telluride, one of triglycine sulfate, and one of gallium arsenide are included in the present study.

The three mercuric iodide crystals were grown by *identical* physical vapor transport *procedures*, one on Spacelab III, a second from *identical source material* under full gravity, and a third also terrestrially from more highly purified material recently available. The resulting crystals were state of the art for each material, as demonstrated by the performance of detectors made from directly comparable material and by the diffraction images.

The two lead tin telluride crystals were grown *by identical Bridgman techniques from identical source material*, one on Space Shuttle STS 61A and the second terrestrially. The images strongly suggest that this material also is state of the art for such a ternary crystal.

The triglycine sulfate crystal consisted of a terrestrial seed and additional growth *by identical techniques from identical solution* on Spacelab III. The images indicate that this material contains relatively few irregularities.

The gallium arsenide crystal consisted of a Czochralski seed and Bridgman regrowth, all terrestrial, but carried out under procedures identical to those due to be employed shortly in a space experiment. The purpose of this particular re-growth experiment is to examine various aspects of the growth rather than to grow immediately the most regular material. The terrestrial material is of interest in its own right for diffraction imaging just now in addition because it is the first Bridgman material to be observed by high resolution diffraction imaging.

## 5. The Images

### 5.1 Mercuric Iodide

#### 5.1.1 Terrestrial Crystal Compared with Spacelab III Crystal

**Figure 1:**
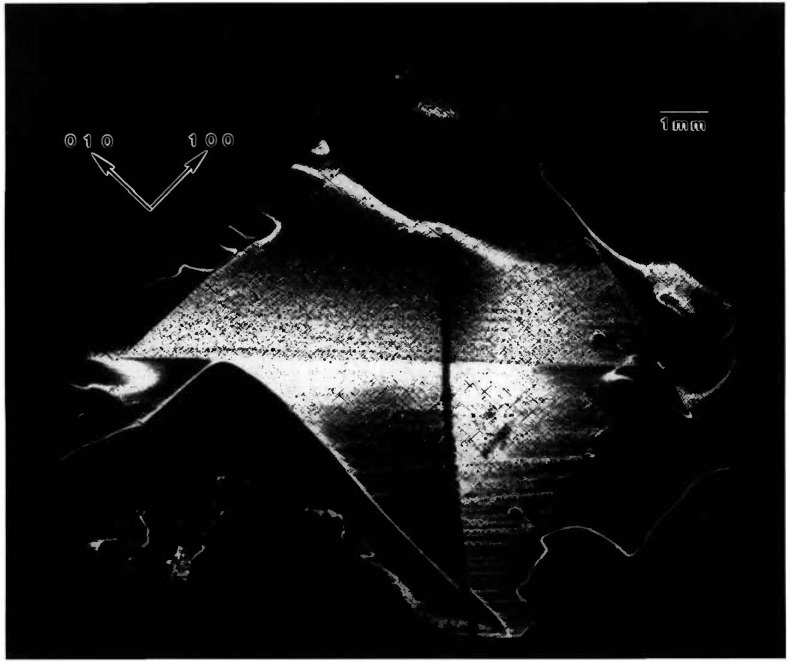
High-resolution (1 1 12) 8 keV diffraction image in Bragg geometry of the (001) surface of terrestrial HgI_2_ crystal comparable to that grown on Spacelab III. Lighter areas diffract more strongly.

**Figure 2:**
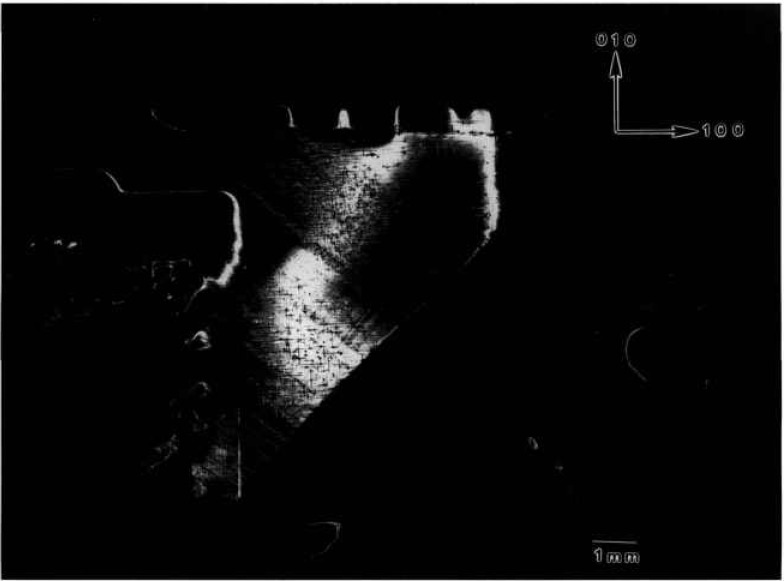
High-resolution (0 1 11)8 keV diffraction image in Bragg geometry of the (001) surface of terrestrial HgI_2_ crystal comparable to that grown on Spacelab III. Lighter areas diffract more strongly.

**Figure 3:**
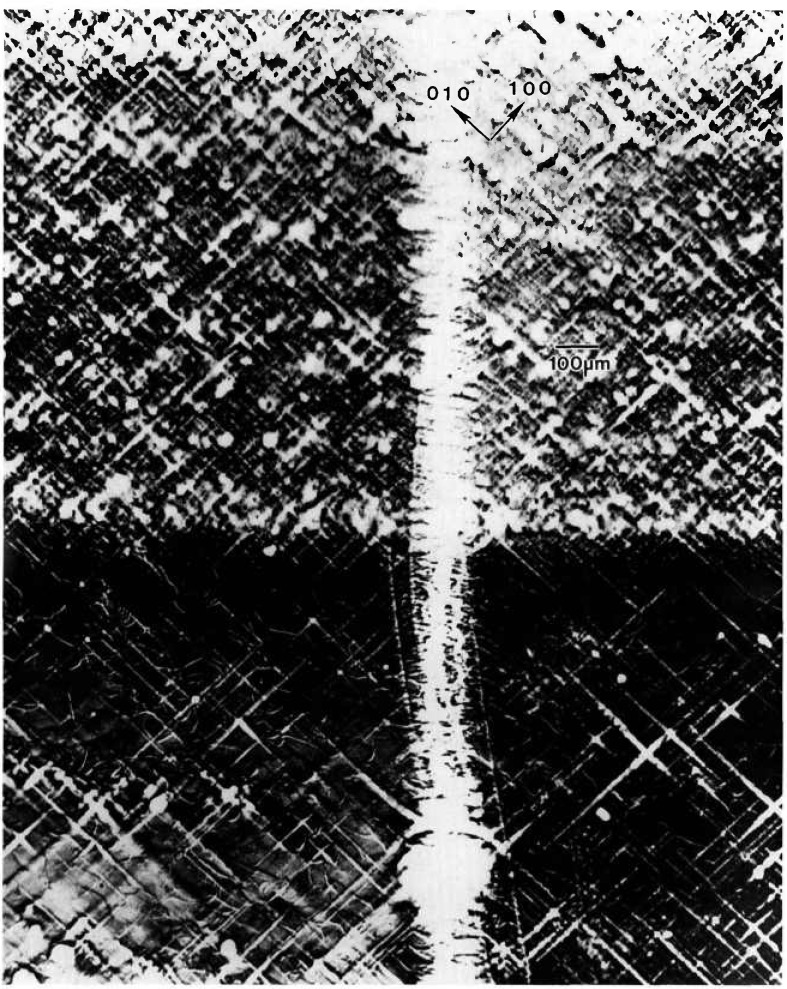
Enlargement of central portion of figure 1, (1 1 12) diffraction. Darker areas diffract more strongly.

While the terrestrial mercuric iodide crystal grown from source material identical to that used for a crystal grown on Spacelab III diffracts over a range of one half degree, a large central portion is sufficiently regular to diffract only within a few minutes of arc. Full high-resolution diffraction images of this terrestrial crystal appear in figures 1 and 2, and an enlarged portion of the first in figure 3.

Most of the central portion of the crystal is in diffraction in the (1 1 12) image in figure 1, indicating lattice regularity with respect to rotation around a [110] axis of the order of a few arc seconds. However, the absence of diffraction in a wide [110] (vertical) stripe in the center of this figure indicates that the lattice is deformed by a sharp twist of about 10 minutes of arc around the orthogonal axis defined by this stripe. The extent of this twist is determined from the 100 μm width of the stripe, and the knowledge that the photographic plate was located about 3.5 cm from the crystal. This twist of the crystal lattice is evident also in the (0 1 11) diffraction image, figure 2, for which the crystal was rotated azimuthally 45°. In this orientation, the misalignment of the two parts of the crystal precludes bringing them simultaneously into diffraction. Examination of a number of full images and of a sequence of real-time images indicates that the principal lattice twist axis itself bends through several minutes of arc, differing slightly in the two subgrains.

The other principal large feature of the full images of this crystal is a set of textural stripes, which are oriented in the $1\bar{1}0$ direction. Enlargements such as that in figure 3 show these stripes to consist of a relatively high density of discrete features, typically out of diffraction in these images and therefore ascribable to one or more additional phases. Some of these features take the form of thin {100}-oriented stripes a few micrometers wide; they are sometimes crossed. The others are more irregular, globular features, 1–60 μm in diameter. These may differ completely from the stripes, but may simply represent similar stripes normal to the (001) image and projected on it. In those regions characterized by a high density of discrete features, diffraction appear to be restricted to small (≈5 μm) cells of the type observed in scanning cathodolu-minescence microscopy [9].

The other areas of the crystal contain similar features that are out of diffraction, but with a much lower density. In addition, however, these regions contain thin, curved features marked by varying sections of higher diffraction, lower diffraction, or alternating regions of higher and lower diffraction in in tandem. The inability to observe diffraction in Laue geometry in the current series of experiments, because of the sample thickness, precludes firm identification now of these features as dislocations.

The nature and arrangement of these various features in this sample make this crystal into a Rosetta stone in understanding the evolution of irregularity in mercuric iodide. One principal question that arises is associated with the origin of the lattice twist. Does it appear during growth or only later during subsequently handling of this very soft crystal? Six distinct observations all indicate that this lattice twist occurred during growth and indeed indicate the growth direction. The first two observations are that the twist axis does not extend across the entire crystal, and that once started, the magnitude of the apparent separation of the two parts of the images does not increase. It is difficult to conceive of such a partial lattice twist, one lying precisely in the (001) plane, developing through inadvertent mishandling. The third observation is that this twist axis is normal to the $\left[1\bar{1}0\right]$ layered texture formed by a high density of additional phase features. These layers appear to be broad striations formed during growth and to indicate its direction; the [110] lattice twist axis appears to be aligned with the crystal growth direction. Fourth, the gradually curved nature of some of the linear multiple phase configurations in the vicinity of the lattice rotation is more consistent with growth than with post-growth bending. Fifth, the onset of the lattice twist immediately precedes a major textural change that appears to be growth-related. The final observation is the bending that has been noted in the lattice twist axis, bending that differs in the two resulting subgrains.

Examination of the interfaces between the widest stratum of high-density features and the adjacent low feature density strata confirms the growth orientation and the origin of the lattice twist. The linear additional phase features in the low-density layer immediately adjacent to the high-density region near the center of the crystal appear correlated closely with individual features in the high-density region. Growth thus took place in this part of the crystal from the high-density stratum to the low-density stratum, i.e., in a direction projecting onto the (001) crystal surface in the $\left[\bar{11}0\right]$ direction. Moreover, as just noted, the sharp lattice twist appears to begin immediately preceding the onset of the broad textural stratum of high density of (precipitate) features, where it joins the preceding low feature density textural stratum.

All of these observations are consistent with a growth model in which growth begins in the extreme [110] corner of this crystal (in fig. 1 this is the top corner) and proceeds relatively uneventfully in the $\left[\bar{11}0\right]$ direction (downward in fig. 1), or in a direction projected onto this direction in the images, until just before the onset of the wide swath of a high density of additional phase features, one of which initiated the sharp lattice twist. The twist then propagated for the remainder of the growth. During this subsequent growth, briefer periods of relatively high additional phase density alternate with periods of relatively low additional phase density.

The nature of the additional phase material is suggested by evaporation of such crystals. As material is removed, small specks of foreign material similar in size to the additional phase features observed in this study accumulate on the surface, at an irregular rate. Chemical analysis indicates these specks are neither mercury nor iodine precipitates but rather consist of organic and metallic impurities with a 70% carbon content and a wide variety of metals. It is tempting to associate these observed impurity formations with the additional phase features observed in diffraction and therefore to conclude that these impurities reside in such crystals in discrete form.

The morphology of the diffraction images permits us to develop two alternative growth models, which tie together all of these observations. Growth over a region of a few micrometers forms a crystal with a relatively high degree of purity and crystal perfection, creating small regions that diffract strongly. Impurities are rejected from the crystal during this stage of the growth process, in a manner similar to constitutional supercooling, and accumulate near the growth surface.

In one model, the level of impurities after growth of a few micrometers accumulates to such an extent that they precipitate out, marking the local growth surface in {100} directions. At reentrant corners of such {100} growth surfaces a globular precipitate possibly forms. In a second model, the rejection of impurity stimulates dendritic growth, which leaves the linear features observed in {100} directions. In this case, the globular form of the precipitate may form in the reentrant dendritic locations. Alternatively, the features that appear to be globular may simply represent the cross section of dendrites normal to the image surface. None of our observations to date permit us to distinguish absolutely between these two models.

Either model involves modulation of the general impurity level by an as yet unidentified process that produces textural stripes or striations delineated by changes in the density of precipitates. The resulting composite formed in either model resists deformation. It consists of relatively pure and thus relatively strain-free components.

#### 5.1.2 Spacelab III Crystal

**Figure 4:**
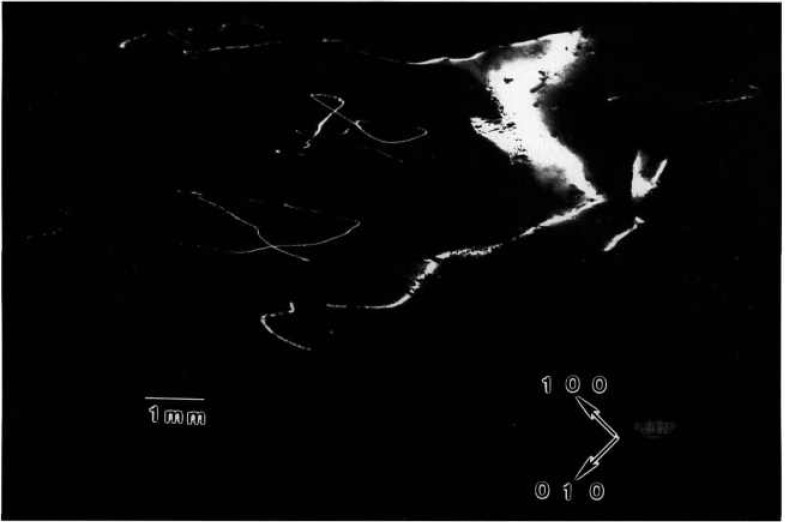
High-resolution (1 1 10) 8 keV diffraction image in Bragg geometry of (001) surface of Spacclab III HgI_2_ crystal. Lighter areas diffract more strongly.

**Figure 5:**
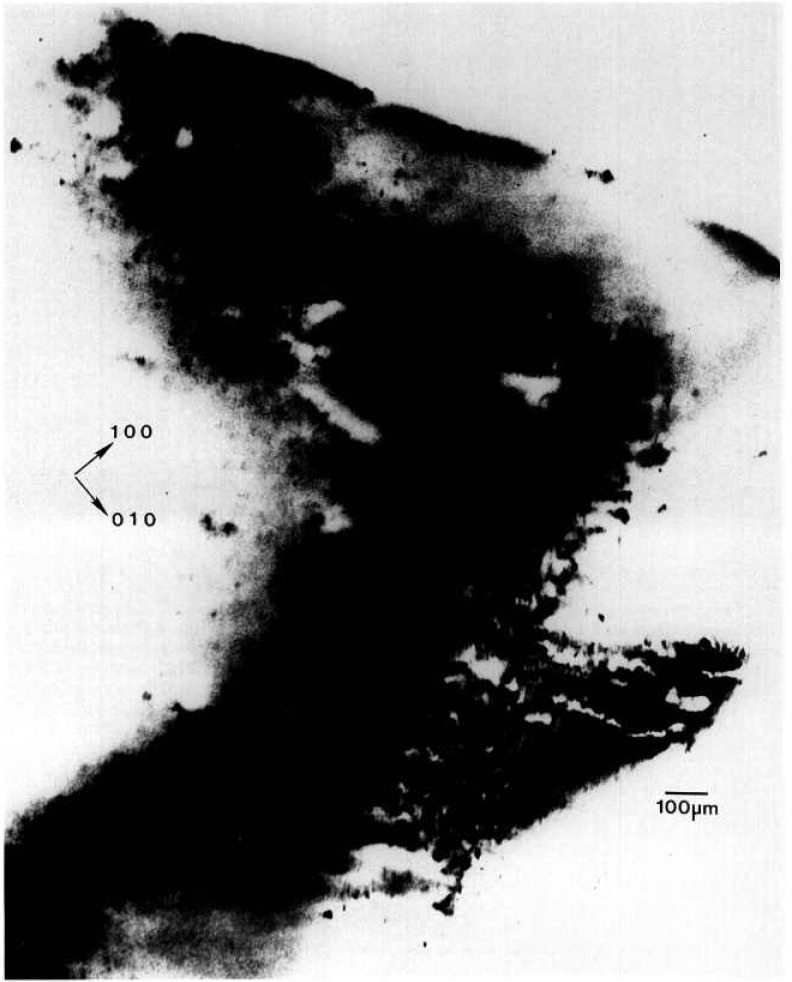
Enlargement of main region of figure 4, (1 1 10) diffraction. Darker areas diffract more strongly.

A crystal grown in Spacelab III from material identical to that used for the terrestrial growth of the crystal shown in the preceding section diffracts over a wider angular range, about one and one half degrees. A full high-resolution diffraction image appears in figure 4 and an enlarged region of this in figure 5. It is clear from the appearance of the full images as well as from the one and one half degree acceptance angle for diffraction that the lattice orientation or parameter of the space crystal in its entirety is less uniform than the comparable terrestrial crystal shown in figures 1–3: that is, less of the space crystal appears in diffraction at a given angle of incidence than does the comparable terrestrial crystal, indicating gradual variation either in lattice parameter or lattice orientation, or both.

Perhaps closely related, but potentially far more important, is substantial reduction in the enlargements of the images of the Spacelab III crystal of arrays of features that are out of diffraction, the textural arrays characteristic of the comparable terrestrial crystal. A few irregular regions of the order of 50 μm across that are out of diffraction are observed, but they are much less pervasive and sharply delineated than are those in the terrestrial crystal. None of the crystallographically oriented regular regions that are typically out of diffraction in the images of the comparable terrestrial crystal are observed in the Spacelab III crystal. The formation of regions of additional phase thus appears to be almost completely suppressed in the crystal grown in microgravity.

The Spacelab III sample differed from the terrestrial sample not only by its growth in microgravity but also by the superposition of graphite electrodes; so that its performance as a neutron and x-ray detector could be measured. Since graphite is relatively transparent to x rays, these electrodes were not expected to interfere with the imaging process itself. While they could in principle have affected the surface strain, we found no evidence for this.

However, this crystal was not encapsulated. With the passage of the 5 years since the growth of this crystal, some deterioration in electronic performance of the device made from it actually has been observed, as is characteristic also of unencapsu-lated devices made in terrestrial environments.

The observed gradual variation in lattice is consistent with varying retention within the lattice of some foreign material. This leads to increased interest in the results of the growth of a mercuric iodide from the much purer material on a future flight.

#### 5.1.3 Terrestrial Crystal to Be Compared to a Future Flight Crystal

**Figure 6:**
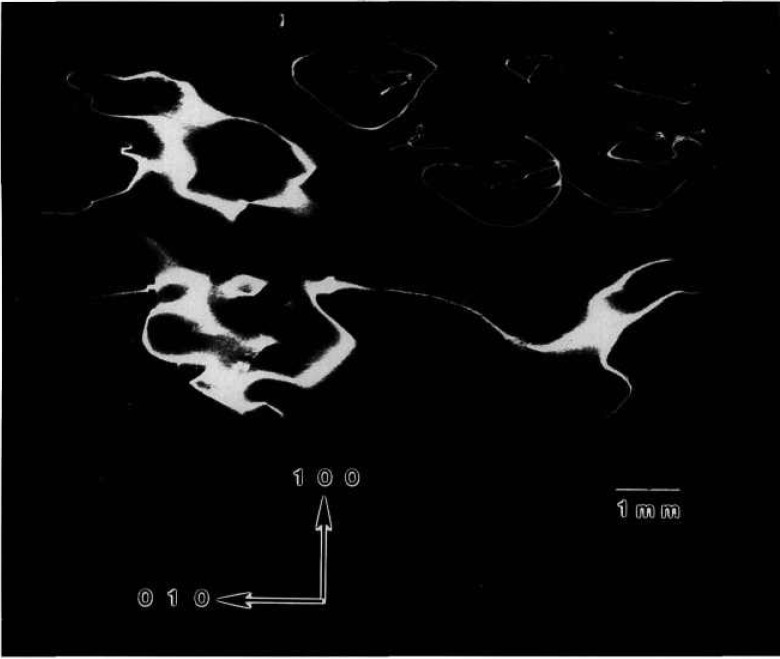
High-resolution (1 0 10) 8 keV diffraction image in Bragg geometry of the (001) surface of terrestrial HgI_2_ crystal, comparable to crystal to be grown on future flight. Lighter areas diffract more strongly.

**Figure 7:**
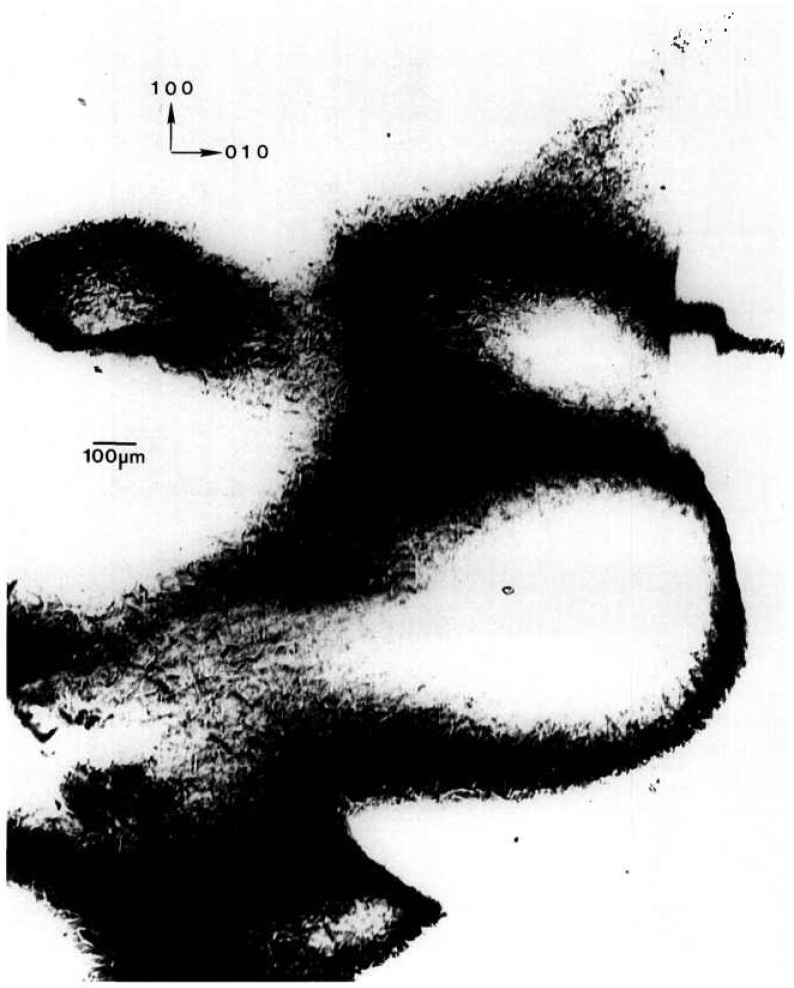
Enlargement of lower left portion on figure 6, (1 0 10) diffraction. Darker areas diffract more strongly.

Models ascribing a central role in the structure and properties of mercuric iodide detectors to impurities are reinforced by observation of a third mercuric iodide crystal, grown terrestrially in an identical manner from higher purity material similar to that to be used on a future flight. It diffracts over a full two degrees. A full high-resolution diffraction image appears in figure 6, with an enlarged region in figure 7. The extent and character of the diffraction in these images, reflecting the general lattice uniformity, resembles much more the diffraction from the Spacelab III crystal than that from its terrestrially-grown counterpart. Moreover, the absence of an array of small features that are out of diffraction also gives these images much more the character of those from the Spacelab III crystal than those from the terrestrial crystal grown about the same time from similar material.

The performance of devices made from the new high purity material approaches the original performance of the device made from the Spacelab III crystal. The improved performance of the Spacelab III crystal is traceable to the higher mobility of its charge carriers. By contrast, however, the purified terrestrial crystal here is characterized by improved carrier lifetime. Although the electronic improvements are quite distinct in these two cases, in neither crystal do we find the additional phase features that we have observed in the first terrestrial crystal. Thus absence of additional phase precipitates appears to be much more important to device performance than the generally higher level of lattice uniformity that we observe in the first terrestrial crystal. The stiffening provided by additional phase precipitates apparently comes at too high a price in terms of charge carrier trapping.

Future space growth of this high purity material now assumes particular interest. Will incorporation of residual impurities in the final crystal even below their currently low level in the charge material be achieved in space growth? And, if so, will these lower impurity levels lead to greater general lattice uniformity? And finally, will this new level of regularity result in still further improvement in device performance, improvements both in carrier mobility and in carrier lifetime?

### 5.2 Lead Tin Telluride

#### 5.2.1 Terrestrial Crystal Comparable to Space Shuttle STS 61A

**Figure 8:**
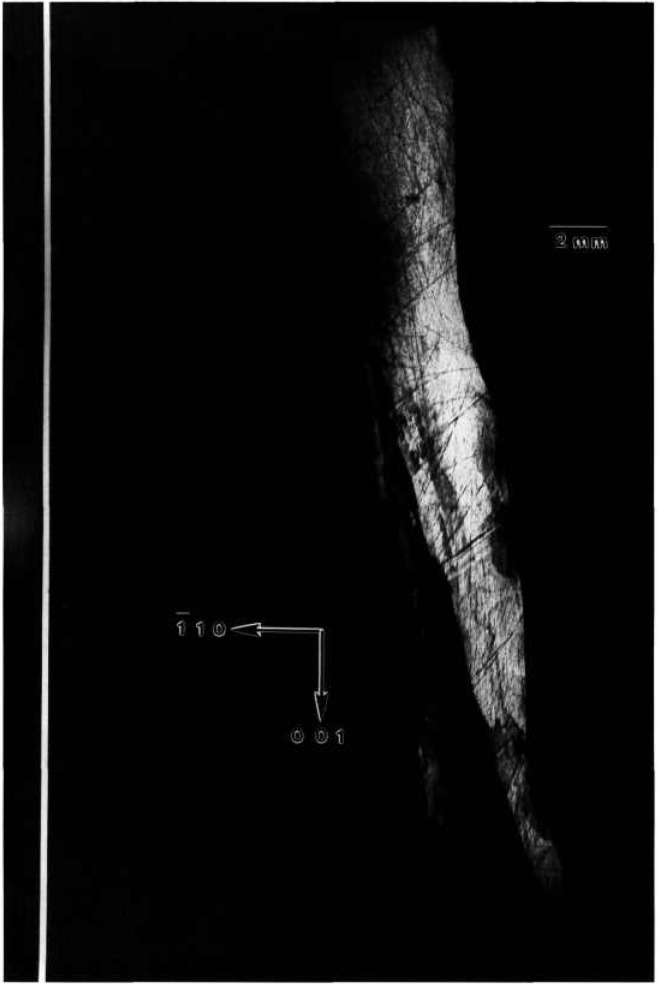
High-resolution (220) 8 keV diffraction image in Bragg geometry of the approximately (220) surface of terrestrial PbSnTe crystal. The growth direction is [001]. Lighter areas diffract more strongly.

**Figure 9:**
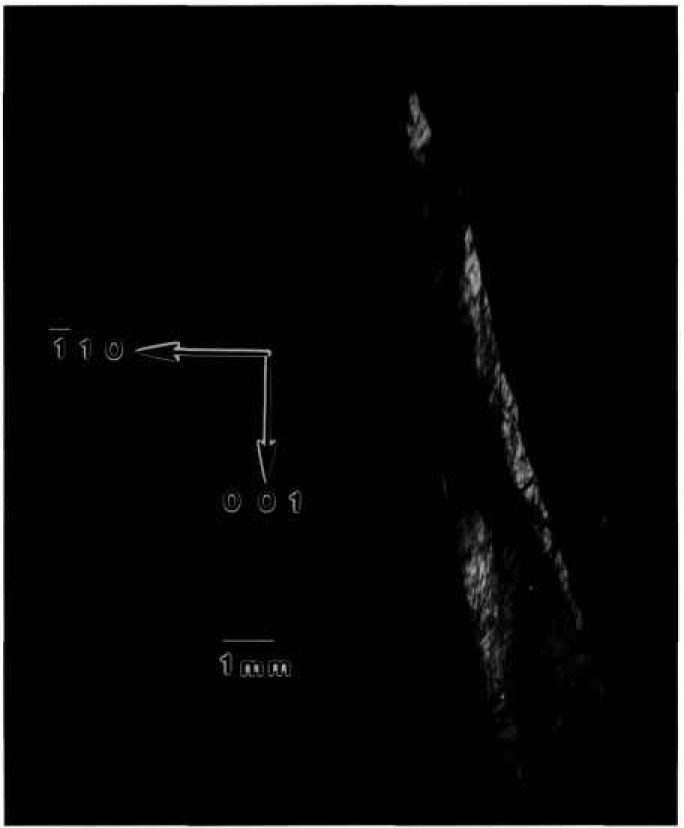
High-resolution (220) 8 keV diffraction image in Bragg geometry of the approximately (220) surface of terrestrial PbSnTe crystal. The angle of incidence is 33 arc minutes larger than that for figure 8; the growth direction is [001]. Lighter areas diffract more strongly.

Crystal Various regions of the terrestrially grown sample of lead tin telluride similar to one grown on Space Shuttle STS 61A diffract as the crystal is rocked over a full two degrees. Full high-resolution diffraction images of two distinct grains are shown in figures 8 and 9. Growth was in the [001] direction, which is oriented “down” in all figures.

The sample was a regular half cylinder. The sharply delineated irregular outlines of the image in figure 8 thus indicate immediately that several grains are present: the curvature of the $\left[1\bar{1}0\right]$ (right hand) edge indicates that a subsidiary grain started to grow almost simultaneously with the main grain. Then, after 1 cm of growth, a third grain started to grow between the center of the boule and the opposite edge of the main grain. It grew laterally more rapidly than the nucleating grain, however, displacing and, after another 2 cm, completely overtaking the growth of the nucleating grain. The new grain is brought into diffraction in figure 9 simply by rotation of the sample about the boule (growth) axis.

Subgrains within each of the main grains are clearly visible through terraced variation in contrast and can be studied in real time images as the crystal is rotated. The generally strong diffraction from a 1.5 cm length of each of the two principal grains observed is notable, however, in light of the increase in tin level from 14 to 18% during the first 3 cm of growth visible in these images. The fractional change in lattice constant over the 1.5 cm length of the grains is 4 × 10^−4^, which changes the Bragg angle by 90 arc seconds. Nevertheless, diffraction is observed in a single image of one of the grains through broadening by kinematic scattering, which is difficult to quantify, as well as by local compositional variation. Because of the mixture of these two broadening mechanisms, unfortunately we can not use the broadening to evaluate the degree of local compositional variation.

**Figure 10:**
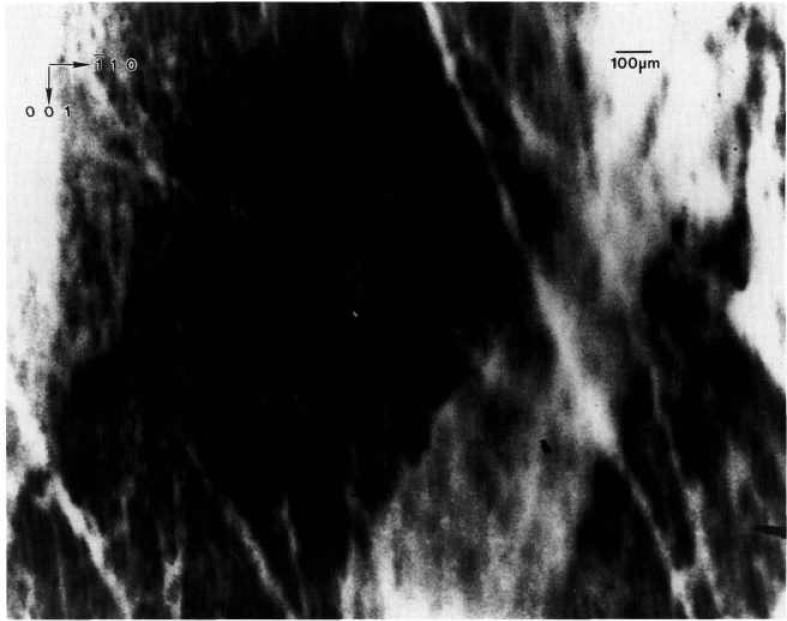
Enlargement of central portion of figure 8, (220) diffraction. Darker areas diffract more strongly.

Other aspects of this variation are evident in enlargements such as figure 10. Cellular regions of high diffraction varying in size from ten to several hundred μm are observed. They are separated by lines of reduced diffraction that are 10–50 μm wide.

Many of these lines at first glance appear to be scratches because of their curvature and random orientation. However, three characteristics typical of surface scratches, such as those visible for example in the gallium arsenide images to which we turn later, are not observed in these linear features. First, the lines vary in width, both from line to line, and even over the length of a given line. In reality these lines separate cellular regions of high diffraction. Second, the boundaries of the lines are very indistinct. And third, contrast reversal is never observed in them. They are invariably out of diffraction over their entire length, even as the crystal is rotated while it is observed in real time. Thus, while we cannot rule out scratches, the linear features here differ markedly in several respects from those of typical scratches in other materials. Moreover, they are not observed in the image of the space-grown sample, whose images follow.

We are thus left with the postulate that the highly diffracting cells are separated by material of another phase. The indistinctness of the boundaries between these regions of differing phase strongly suggest gradual change in chemical composition on a scale of 1–10 μm or so, in contrast to the sharp delineation between diffracting and non diffracting features in the images of mercuric iodide discussed above.

The pseudobinary phase diagram along the leadtin axis predicts complete miscibility [11]. However, the observation of similar structure following electrolytic etching led earlier to a series of experiments on the metal/tellurium ratio, which delineated its importance in the growth of this material. This earlier work provides a satisfactory model for the current observations as well [12, 13]. While the metal constituents are widely recognized to be interchangeable, a single phase is preserved only with tellurium concentration in excess of 51%. Below this value, two phases are formed, differing in metal/tellurium ratio. Since the tellurium concentration of the current crystals is 50.1%, two phases are actually to be expected. Constitutional supercooling may also play an important role, depending on the temperature gradients imposed [14].

#### 5.2.2 Space Shuttle STS 61A Crystal

**Figure 11:**
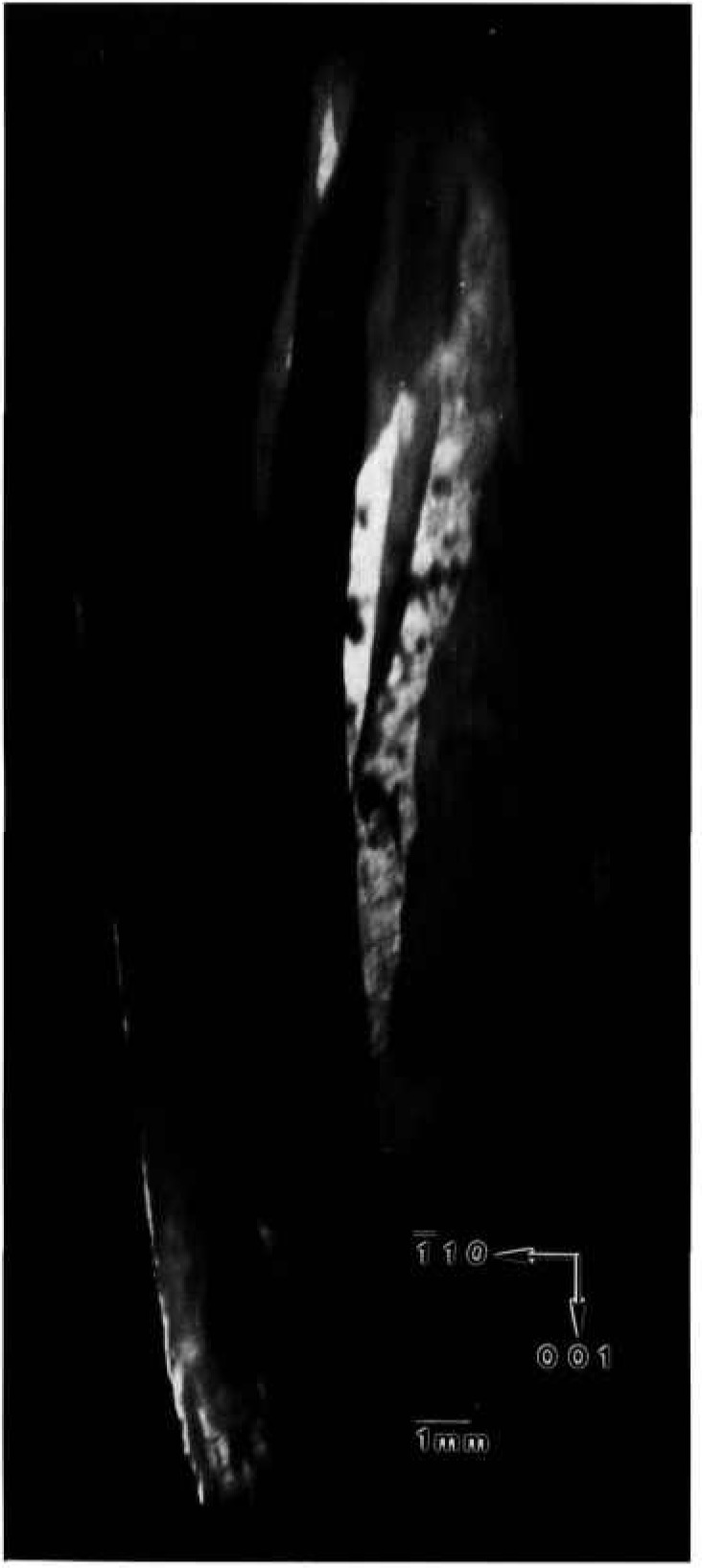
High-resolution (220) 8 keV diffraction image in Bragg geometry from the approximately (220) surface of PbSnTe crystal grown on STS 61A. The growth direction is [001]. Lighter areas diffract more strongly.

**Figure 12:**
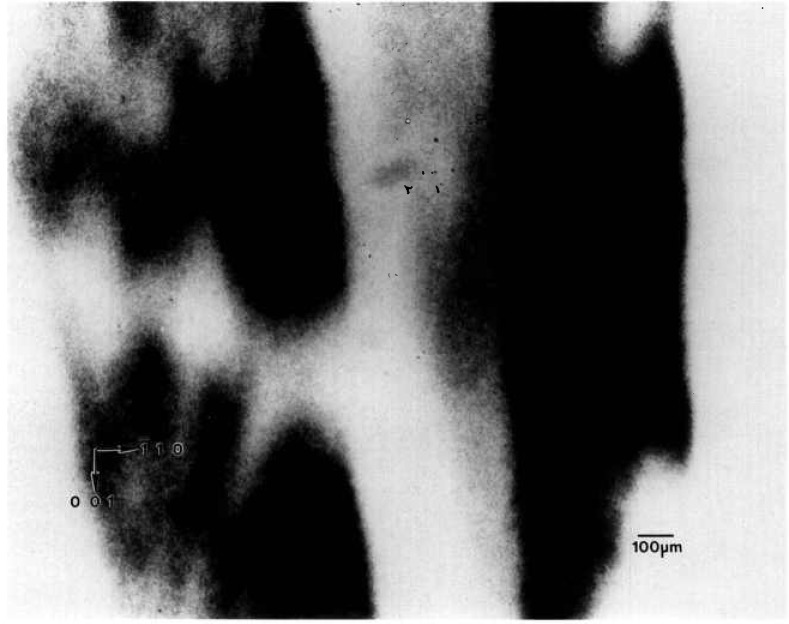
Enlargement of central portion of figure 11, (220) diffraction. Darker areas diffract more strongly.

A full image of a crystal grown on flight STS 61A appears in figure 11 and an enlargement of the central portion of this in figure 12. The multigrain nature of the STS 61A crystal is generally similar to that of the terrestrial crystal. But, while these images appear qualitatively similar to the full images for the corresponding terrestrial crystal, they differ in important ways.

Each grain is more generally uniform than those of the terrestrial crystal. This uniformity follows a drastic reduction in the incidence of linear features and subgrains. As a result, variation in diffraction on a scale of 10–100 μm is greatly reduced. Thus, while the granular structure resembles that for the terrestrial crystal, variation within individual grains from the intrusion of a distinct second phase appears to be suppressed in microgravity. The absence of thermo-solutal instability for this system in microgravity was noted in the preceding section.

### 5.3 Triglycine Sulfate

**Figure 13:**
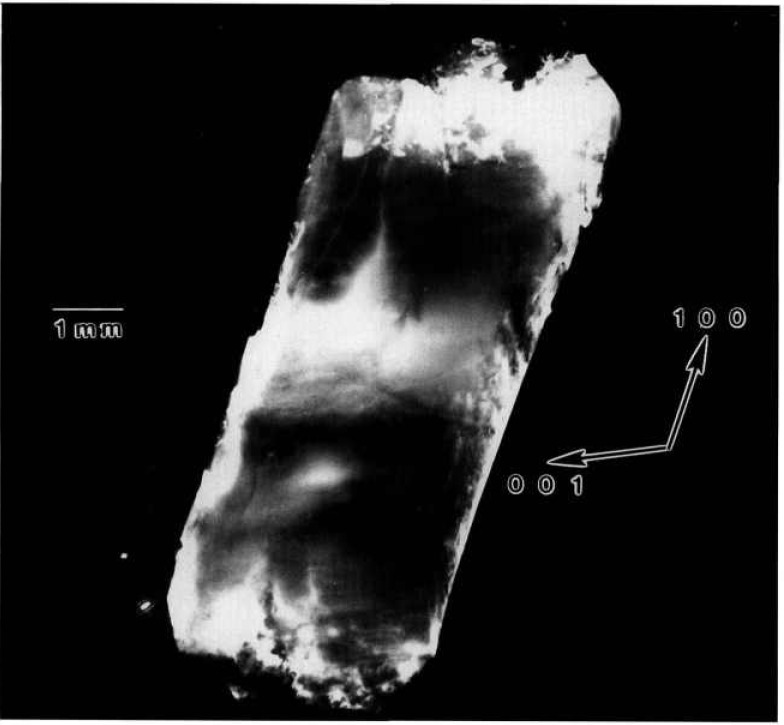
High-resolution (300) 10 keV diffraction image in Laue geometry from the (001) surface of grain C2 of TGS crystal from Spacelab III. Lighter areas diffract more strongly.

**Figure 14:**
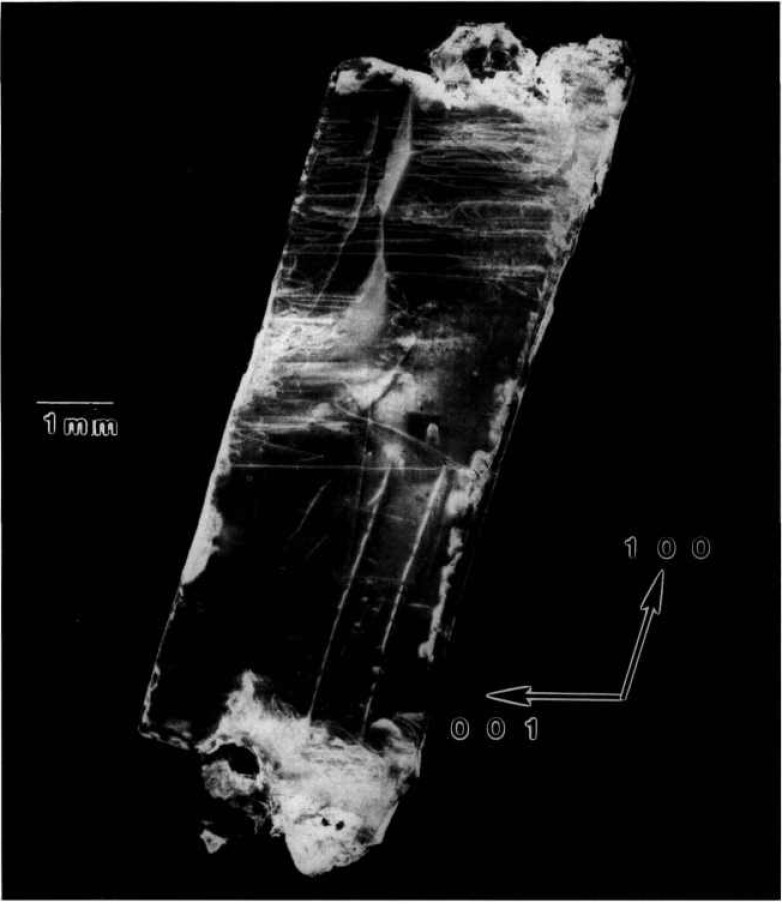
High-resolution (200) 10 kcV diffraction image in Laue geometry from grain B1 of TGS crystal from Spacelab III. Lighter areas diffract more strongly.

**Figure 15:**
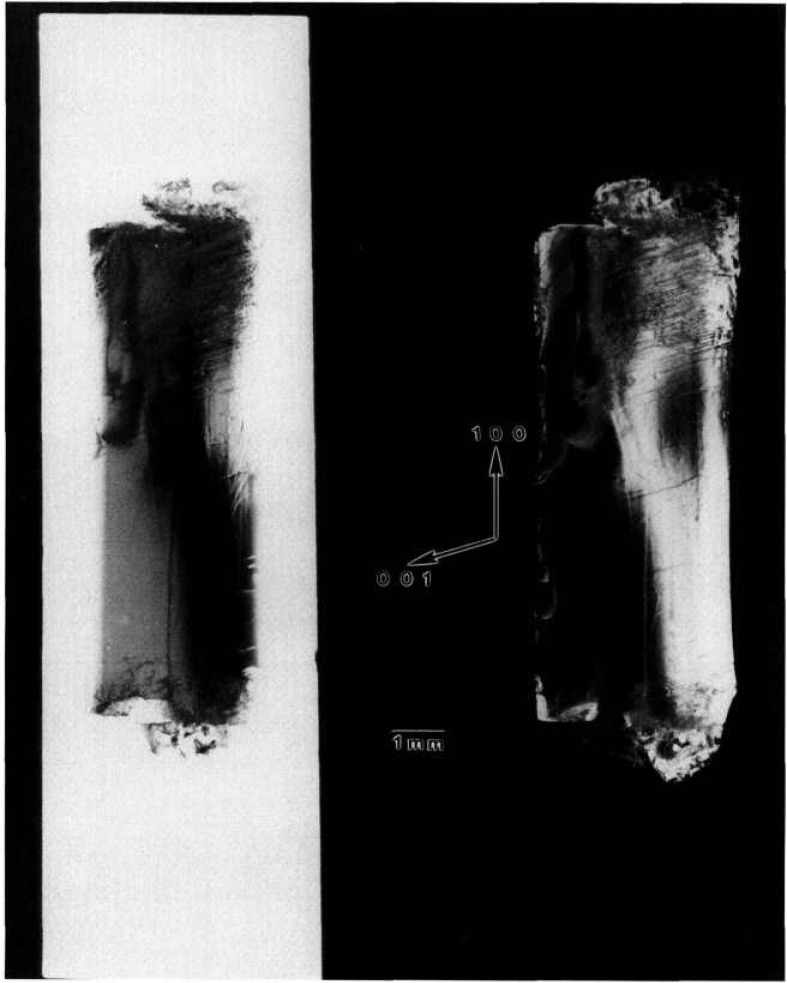
High-resolution (001) 10 keV diffraction image in Laue geometry from grain A2 of TGS crystal from Spacelab III. Lighter areas diffract more strongly.

**Figure 16:**
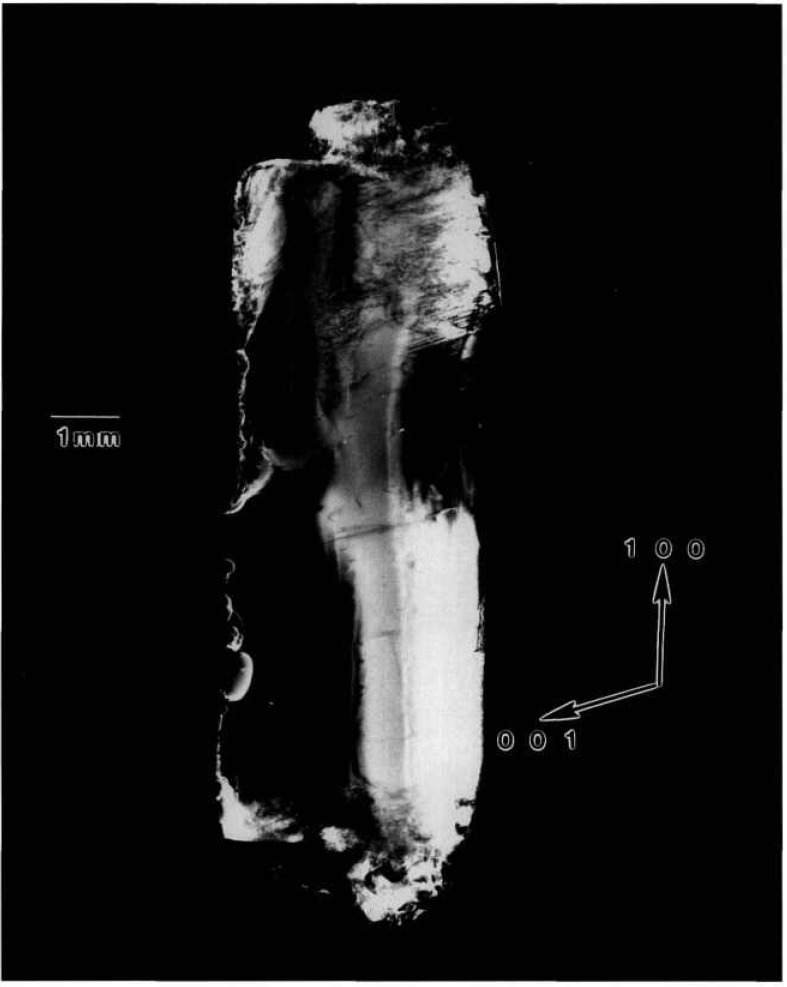
High-resolution (001) diffraction image in Laue geometry from grain A3 of TGS crystal from Spacelab III. Lighter areas diffract more strongly.

A normal slice from a disc-shaped terrestrial seed crystal with additional growth achieved on Spacelab III diffracts into images, each of which appears over less than half of an arc minute. Full high-resolution diffraction images appear in figures 13–16. The character of the diffraction from this crystal is very different from that of the others. This crystal was thin enough and low enough in atomic number to allow diffraction in Laue geometry. Moreover, superimposed images of this crystal taken as it was rotated about its [100] and [001] axes appear in closely spaced groups, each associated with the diffraction directions expected for diffraction from one set of (*h*00) or (00*l*) planes, respectively. The various images have similar, but not identical, shapes. Subsequent work, summarized in table 1, indicates that the members of a given group of images appearing at nearly similar diffraction angles come into diffraction at differing sample orientation.

The appearance of images in groups indicates that this crystal consists of layered grains whose lattices are similar but rotated with respect to one another by rotation about the [100] and [001] axes. Since each image is ostensibly nearly “complete,” the grain boundaries are roughly parallel to the (010) crystal surface. In an optically thick material, transmission through such a layered crystal would be precluded by the misalignment of the successive grains. However, this crystal is optically thin, permitting the observation of symmetrical diffraction from each of the grains in turn. From the occurrence of similar features in pairs of images, which can be ascribed to features shared by adjacent grains at their interface and the degree of clarity, we can assign a tentative order to the various grains as intersected by the x-ray beam. This is the order in which their images are presented in the figures and in table 1. Most of the features thus appear to be associated with irregularities at the granular interfaces, although radiographic effects from each layer are present.

The seed portion of this crystal takes upmost of each image. Space growth was in the $\left[00\bar{1}\right]$ direction along that one edge of the seed. The absence of a clear demarcation between the seed and new growth in this region in most of the images is in contrast to the terrestrial growth of comparable material. The interface between the seed and the new growth is visible in figure 14, but irregularities in this grain do not appear to propagate into the new growth in the central portion of the disc. In the other grains, irregularities from the seed indeed appear to have propagated into the part grown in microgravity. Toward the edge of the disc, the irregularity observed in all of the grains is consistent with rapid growth anticipated from the defects observed near the edge of the seed. Irregularities near the edge in such systems before faceting becomes fully developed later in growth are observed optically as well.

The last of these images, figure 16, differs in shape along the growth edge from the others. It thus appears that new growth did not occur uniformly on all layers. On this one layer, growth appears to have been much slower than on the others, although this may represent initial etching of the seed crystal associated with premature contact with the solution.

### 5.4 Gallium Arsenide

**Figure 17:**
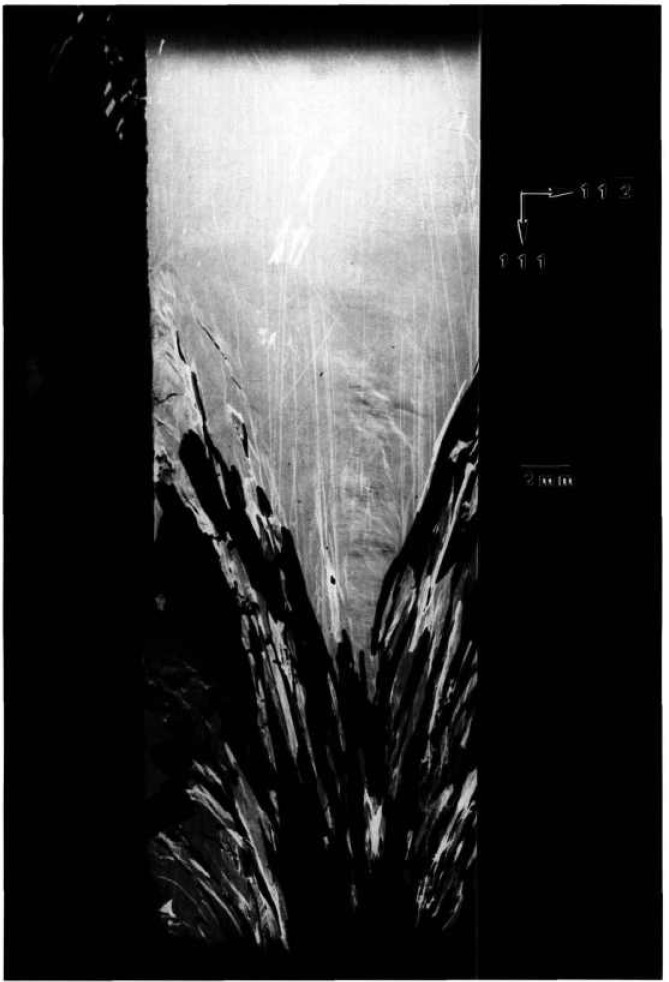
Low resolution (220) 8 keV diffraction image of the approximately (220) surface of terrestrial GaAs crystal, in Bragg geometry. The sample was rocked through an angle of 4° during this exposure; the growth direction is [111]. Lighter areas diffract more strongly.

**Figure 18:**
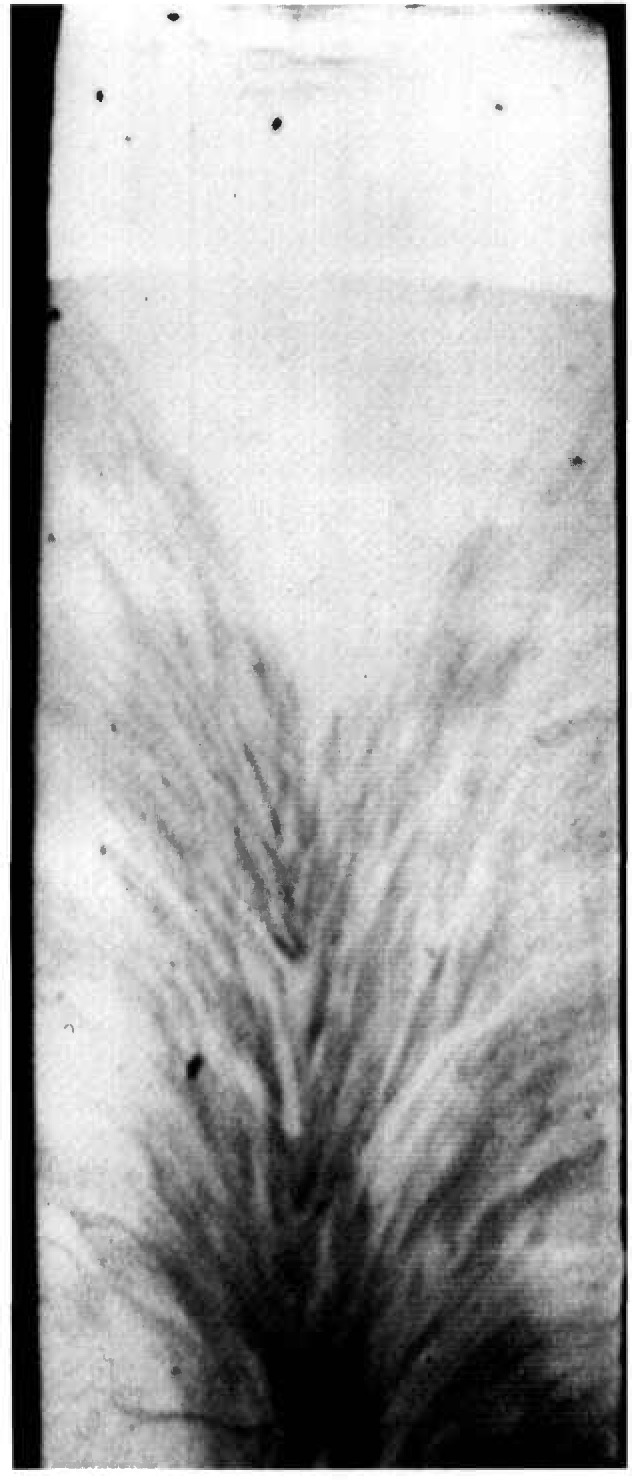
Infrared image of crystal shown in figure 17.

**Figure 19:**
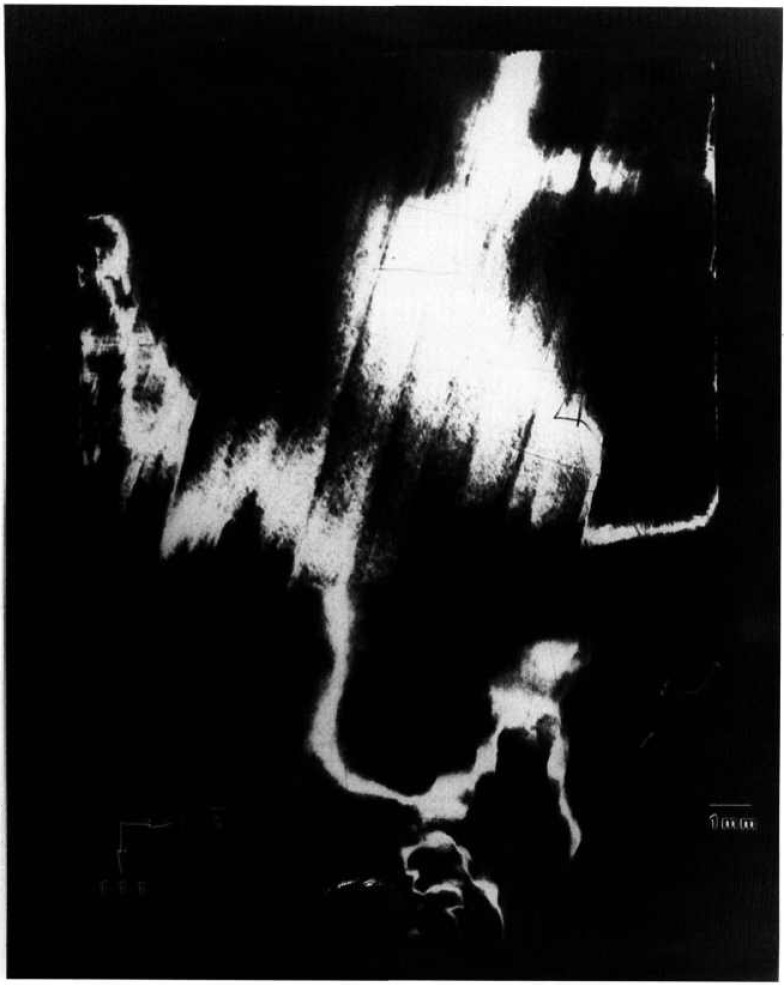
High-resolution (220) stationary diffraction image from the approximately (220) surface of terrestrial GaAs erystal in Bragg geometry. The growth direction is [111]. Lighter areas diffract more strongly.

**Figure 20:**
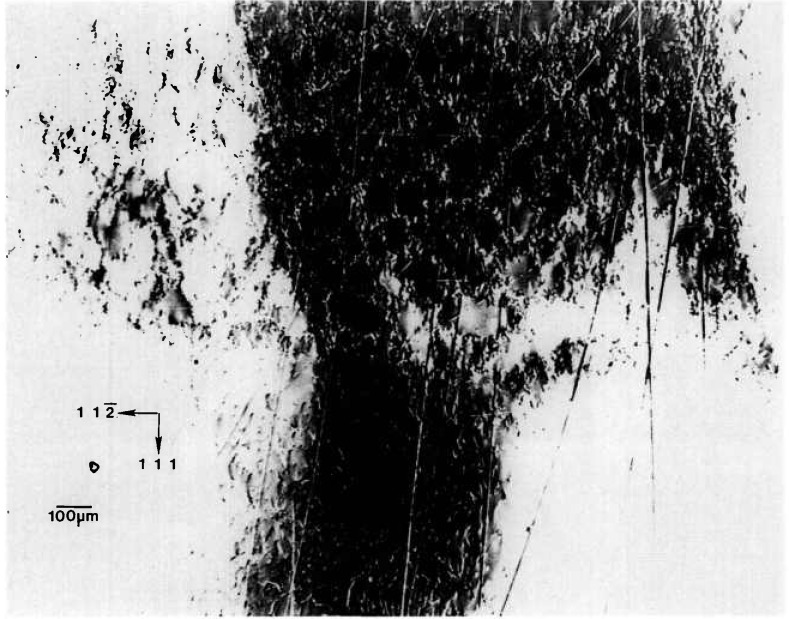
Enlargement of figure 19, (220) diffraction, near the center of the seed/new growth interface. Darker areas diffract more strongly.

A terrestrial crystal of lightly selenium-doped Bridgman grown gallium arsenide diffracts over several degrees. Growth of identical material by identical techniques is scheduled for an early space flight. A low resolution diffraction image of the terrestrial crystal, achieved by rocking the crystal 4° around a $\left[11\bar{2}\right]$ axis during diffraction is shown in figure 17. An infrared image of the same crystal is shown in figure 18. The similarity of these two images is striking. The additional information in a full high-resolution diffraction image of this crystal, figure 19, is evident. An enlargement of a portions of this image is shown in figure 20.

The demarcation of the Czochralski seed from the new Bridgman growth is very clear in those images in which this region is in diffraction. The seed/growth boundary is delineated in two ways. First, toward the periphery of the boule it marks a smooth limit to diffraction, past which the lattice does not diffract under the same conditions. Thus, either the lattice constant, or orientation, or both differs in the new growth. Second, in the one region of the seed interface supporting diffraction from both sides, the mesoscopic structure of the growth is observed to be transformed at the interface. The cellular structure of the seed is characteristic of diffraction images of Czochralski-grown undoped gallium arsenide. In the new Bridgman growth, the formation of cells appears to be completely suppressed. Freedom from other demarcation, however, indicates that, to the extent permitted by the lattice parameter match, the two lattices continue uninterrupted. The lattice parameter mismatch appears to set up a gradual warping of the crystal lattice. Further analysis of the features observed is precluded by the inability to observe diffraction in Laue geometry.

## 6. Summary of Initial Observations

These results are summarized in the following sections. The three crystals of mercuric iodide, two of lead tin telluride, one triglycine sulfate crystal, and one gallium arsenide crystal show remarkable differences in their irregularities.

### 6.1 General Effects of Microgravity

Seven very different crystals do not provide a sample that is sufficiently large to form definitive conclusions. Nevertheless, our observations provide guidance for further evaluation and crystal growth. The formation of pervasive multiple phases observed in two terrestrial crystals appears to have been greatly suppressed on growth of two comparable crystals in microgravity.

### 6.2 Mercuric Iodide

A terrestrial specimen comparable in growth procedure and source material composition to one grown on Spacelab III displays more than one phase. The features containing the additional phase appear to be globular in part and partly in the form of thin layers oriented along the {100} crystallo-graphic directions of the matrix. One of these features appears to have initiated a sharp lattice twist by 10 minutes of arc around an axis aligned with the growth direction and to have stiffened the two resulting subgrains. Formation of the additional phase material is suppressed both in a comparable Spacelab III crystal and a recently grown high purity terrestrial crystal. At the same time, the general regularity of the lattice of these latter crystals is lower than in the earlier terrestrial crystal. Superior performance of detectors made from these materials thus appears to be limited far more by sharp discontinuities associated with additional phase(s) than by slow variation in the lattice.

### 6.3 Lead Tin Telluride

The mesoscppic structure of lead tin telluride appears also to be influenced strongly by the intrusion of additional phase material. But in this instance all available evidence points to identification of the additional phase with the major constituents. Indeed, the presence of two phases has been predicted for systems with tellurium concentration very close to 50%.

Although the STS 61A crystal has grain structure that appears to be similar to that of the comparable terrestrial crystal, the formation of the subgrain variation characteristic of the terrestrial sample is suppressed in microgravity. This suppression is correlated with the predicted thermo-solutal stability in microgravity.

### 6.4 Triglycine Sulfate

Interpretation of the space-growth of triglycine sulfate has been complicated by the layered structure that we observe in the seed. Defects in one of these layers appear not to have propagated in the central portion of the disc in microgravity, while defects in the other seed layers appear indeed to have propagated into the new growth. In addition, one of the seed layers appears to have grown at a rate slower than the others, but this may simply represent inadvertent contact between the seed and the solution prior to space growth.

In the IML 1 mission scheduled, use of multi-faceted natural seeds is planned. They will be characterized not only for various physical properties but also for defects and structural properties prior to flight. In this way, definite information should be obtained on the generation and propagation of defects during growth.

### 6.5 Gallium Arsenide

Although gallium arsenide has not yet been grown in space in the NASA program, space growth directly comparable to that used for our terrestrial sample is scheduled shortly. Meanwhile, the mesoscopic structure of our terrestrial Bridgman regrowth has been observed to differ from that of the original Czochralski-grown material. The Bridgman-grown lattice also appears to be warped more than that of the Czochralski-grown seed.

## Figures and Tables

**Table 1:** Layers on sample of triglycine sulfate

Layer	Figure No.	(*hkl*)	Orientation (°)
C2	13	300	+ 6.6
B3		200	+ 2.1
B1	14	200	+ 2.5
A2	15	001	−19.2
A3	16	001	−36.8
